# The Medical Department of the Army

**Published:** 1906-03

**Authors:** 


### Abstracts and Selections.

### The Medical Department of the Army.

The readers of the *Medical Sentinel* scarcely need figures to convince them that the medical department of our army is entitled to all the support it can receive in its efforts to raise the standard of its service. Yet, so important is this question, that we may be pardoned if we quote figures based upon the official records. There has rarely been a conflict of any duration in which at least four men have not perished from disease, for every one from bullets. In the Russo-Turkish war, 80,000 men died from disease and 20,000 from wounds. In the Crimean campaign it is asserted on eminent French authority that in six months the allied forces lost 5,000 soldiers from disease, and only 2000 from casualties. In the French campaign in Madagascar in 1894, of the 14,000 men sent to the front, 29 were killed in action, and 7000 succumbed to disease, most of which was preventable. In our Spanish-American war in 1898, in a campaign the actual hostilities of which lasted six weeks, the deaths from casualties were 293, while those from disease were 3681, or nearly 14 to 1.

Perhaps some people might have thought, up to the time of the Russo-Japanese war, that such mortality from disease was unpreventable. But the Japanese have shown us that this view of the matter is not the correct one. Medical men knew this before, but if the public had become quick to become awake to the facts, their representatives in Congress would not have been permitted to go to this late day without enacting laws that would give the required relief. From February, 1904, to May, 1905, there were 52,946 deaths in the Japanese army from casualties and wounds, while the total deaths from sickness amounted to 11,922. This record is unparalleled and unapproached in the history of warfare, and merely shows what can be accomplished by intelligent and adequate medical organization. One writer, in answering the question as to how this was accomplished, says it was done in three pre-eminently fundamental ways. First, thorough preparation and organization for war such as was never before made in history, as to the medical department; second, through the simple, non-irritating, easily digested ration furnished the troops; and third, because of the brilliant part played by the members of the medical profession in the application of practical sanitation and the stamping out of preventable disease in the army. Japan organized the medical department of her army on broad, generous lines, and gave its representatives the rank and power their great responsibilities merited, recognizing that they had to deal with a foe which history has shown has killed 80 per cent of the soldiers who have died, in other wars. As this author pertinently remarks: "She even had the temerity (strange as it may seem to an American or English army officer) to grade her medical men as high as the officers of the line, who combat the enemy who kills only 20 per cent, and to accord them equal authority, except, of course, in the emergency of battle, when all the authority devolves, as it should, on the officers of the line. In her home land she organized the most splendid system of hospitals that has ever been devised for the treatment of the sick and wounded, and with her army at the front, she put into execution the most elaborate and effective system of sanitation that has ever been practiced in war."

It is a sad pity that the near relatives of the unfortunate soldiers who die of preventable diseases, can not be organized, so that they might with greater force demand that their representatives in Congress amend the present inadequate laws. When deemed necessary, tens of thousands of dollars will be expended, and that properly, by the State, in the prosecution of a criminal who has committed one murder. The theory of the prosecution is that life is the most precious possession of man. And yet where not one victim is involved, but tens of thousands, appropriations and salient measures are withheld, because a few paltry tens of thousands of dollars are involved. Our soldiers, in times of war, are slaughtered by disease by the thousands. After the turf is laid upon their last resting place, a headstone is placed to mark the spot, the world moves on as before, nobody is punished, and no united effort is made on the part of mourning relatives to secure a reform. The father sheds a tear over the body of his promising son, a victim of typhoid fever. He has, perhaps, no other son to give to the remorseless juggernaut of disease; or he fondly hopes, if he has other sons, that no call for their services will be made by the country. The mother, who has, perhaps, lost her sole support, has no voice in the affairs of the nation, and would not know how to exercise it to advantage if she had such a voice. The newspaper reader who learns of the horrible decimation in the camps that have been selected by army officers, without consulting the medical department, and often against their ineffectual protests, is shocked for a few brief days, and acts as if disease were a visitation of God, for which there was no remedy, and no means of prevention.

In the meantime, the medical department of the army, staggered and disheartened by the inadequacy of the law, are still striving, year after year, to remedy the present unfortunate state of affairs. They realize that the conditions existing in the United States, while no worse, perhaps, than in some other countries, are, as regards effective organization, deplorably behind Japan, and disgracefully behind what they should be in such an enlightened country as this. The medical profession of the country can do much to secure an improvement of the status of the medical department of the army. Individual members of the profession can reach their congressmen and senators, whose interest would be valuable. Where they are lukewarm, their interest should be awakened. When war comes, this is a matter of more than general interest; it touches every locality.

The army today is officered for a strength of 100,000 men except the medical department, which is only sufficient for 42,000. As the Surgeon General has succinctly put the case: "The three primary duties of the medical department are (1) to preserve the effective strength of armies by military sanitation; (2) to care for the sick and wounded; (3) to conduct the administrative work of the department. To carry out these objects requires a highly specialized and complex organization, and a numerous trained personnel. Military sanitation is now recognized to be a well marked specialty in medicine, of which the average practitioner knows little more than he does of the methods of military medical administration. The second duty is that for which civilian physicians can be used to advantage, while the first and third must be, in the main, in the hands of trained medical officers in order to secure efficiency, and to relieve the volunteer medical officer of the 'red tape' which, while a necessary evil, is so burdensome and incomprehensible to men not trained to government methods."

The bill which is now before Congress provides for an increase of medical officers from 320 to 450, exclusive of the Surgeon General. It reduces the length of service required in the grade of lieutenant before promotion to captain, from five to three years, in order to correspond with the requirements of the naval service. It increases the proportions in the higher grades to that existing for many years prior to the reorganization of 1901, and nearly equalizes them with those existing in the medical corps of the navy. It provides examinations to determine fitness for promotion up to include the grade of colonel. It provides a medical reserve corps which shall constitute an eligible list of medical officers who shall have been examined, and commissioned if found fit, for service as medical officers with the rank of first lieutenant in case of war or other emergency. These reserve medical officers are intended to replace the contract surgeons, upon which the medical department depends for expansion when its numbers are found to be inadequate for the needs of the service, as, for example, when the army is raised to its maximum authorized strength of 100,000 men. They are not intended to in any way replace the volunteer medical officers.

President Roosevelt is fully alive to the importance of this matter, and in a message to Congress last January, he said: "Not only does a competent medical service, by safeguarding the health of the army, contribute greatly to its power, but it gives to the families of the nation a guaranty that their fathers, brothers and sons who are wounded in battle or sicken in camp shall not only have skilled medical aid, but also that prompt and well ordered attention to all their wants which can come only by an adequate and trained personnel. I am satisfied that the medical corps is much too small for the needs of the present army, and therefore very much too small for its successful expansion in time of war to meet the needs of an enlarged army, and in addition to furnish the volunteer service a certain number of officers trained in medical administration. * * * If the medical department is left as it is, no amount of wisdom or efficiency in its administration would prevent a complete breakdown in the event of a serious war."—*Editorial in Medical Sentinel,* Portland, Ore., December, 1905.

---

Mr. Bok's Bill.—

AN ACT

TO REGULATE THE MANUFACTURE AND SALE OF "PATENT" AND "PROPRIETARY" MEDICINES.

*Be it enacted by the Legislature of the State of................*

Section 1. Each package, bottle, box or other parcel containing what is commonly known as a "patent" or "proprietary" medicine of any kind or in any form, intended for internal consumption by human beings, other than a medicine specially compounded on the written order or prescription of a physician duly authorized to practice his profession in this State, or which shall be hereafter manufactured within this State, or which shall be hereafter manufactured without this State and exposed or offered for sale, or sold or given away, or otherwise disposed of, within this State, shall have both on the outside wrapper of such package, bottle, box or other parcel, and also on the label affixed to such package, bottle, box, or other parcel, in plain English, printed in black letters on white paper, of a size not smaller than of type eight point, so-called, a complete schedule showing all the ingredients contained in such "patent" or "proprietary" medicine, and the exact proportions of each ingredient thereof.

Sec. 2. Whenever any such "patent" or "proprietary" medicine shall contain more than 8 per cent of ethyl alcohol, or more than one-twenty-fifth of 1 per cent of morphin, heroin, cocain, or of the salts or equivalents or derivatives of the same or any of them, or more than one-fourth of 1 per cent of chloral hydrate, or any quantity of belladonna, cotton-root, ergot, or other abortifacient, there shall be printed in plain English, in red letters of a size not smaller than eight point, so-called, on white apper, in addition to the schedule of ingredients hereinbefore required, both on the outside wrapper of the package, bottle, box or other parcel containing the same, and also on the label affixed to such package, bottle, box or parcel, a notice reading as follows:

"This package (or bottle or box or parcel, as the case may be), contains (here give the name and proportion or percentage of the drug, as the case may be), and is therefore under the act of the Legislature of the State of........................marked

"POISON,"

and also the single separate word "poison," which shall be printed separately on a line by itself, in bold-face type, and in letters not less than one-quarter of an inch high.

Sec. 3. The Board of Health of this State is hereby empowered, immediately on the passage of this act, and from time to time thereafter, to make, or cause to be made, a chemical analysis of "patent" or "proprietary" medicines manufactured, or exposed or offered for sale, or sold or given away, or otherwise disposed of, within this State, for internal consumption by human beings, other than those specially compounded on a physician's written prescription as aforesaid. If any such analysis shall show that there has been with respect to any such "patent" or "proprietary" medicine, a failure to comply with the requirements of this act, said board shall at once notify the district attorney of any county in this State in which the said "patent" or "proprietary" medicine is manufactured, or exposed or offered for sale, or sold or given away, or otherwise disposed of, whose duty it shall be to prosecute the person, firm or corporation so violating the provisions hereof.

SEC. 4. Any changes, either in the ingredients or in the proportions or percentages of the ingredients in any such "patent" or "proprietary" medicine manufactured within this State, shall be at once reported by the manufacturer thereof to the Board of Health of this State.

SEC. 5. Any person, firm or corporation who shall manufacture, or expose or offer for sale, or sell, or give away, or otherwise dispose of, any such "patent" or "proprietary" medicine within this State, in violation of the provisions of this act, or any of them, shall be guilty of a misdemeanor and on conviction thereof shall be punishable therefor by a fine of not less than fifty dollars ($50) nor more than five hundred dollars ($500), or imprisonment for not less than thirty (30) days nor more than six (6) months, or both.

SEC. 6. All acts or parts of acts inconsistent herewith are hereby repealed.

SEC. 7. This act shall take effect on the........day of............ 1906.

Commenting on the bill, the *Journal A. M. A.* says: "The provision limiting the maximum strength of morphin and its derivatives to one twenty-fifth of 1 per cent would represent a morphin strength equivalent to one grain in 44 fluid drams (0.04 gm. in 100 c.c.). The average teaspoonful is equivalent to 80 minims (5 c.c.) instead of one fluid dram, and it would contain about 1/32 grain of morphin. This is about what many of these medicines now contain; one of the largest used and which has to be labeled "Poison," in England, contains little more morphin than this.

The manufacturers of soothing syrups, cough syrups, "baby's friend," etc., could easily reduce the morphin amount to place their medicines beyond the law, with an increased consumption and sale as the only result. The quantity allowable should be reduced at least one-half—or, in other words, to one-fiftieth of 1 per cent —but preferably no medicine containing opium, morphin, cocain or their salts or derivatives in any proportion should be exempt; the "Poison" label should be used always when any of these drugs are contained in the preparation.

For the control of nasal hemorrhage tampons can be readily prepared as follows: A layer of cotton is wound around a penholder or similar object until the desired thickness is obtained and then withdrawn. The cotton cylinder is then moistened, squeezed dry and inserted into the nasal cavity. If the projecting end of the tampon is now moistened it will swell up and thus produce sufficient compression.—*International Journal of Surgery.*

